# Using time series analysis approaches for improved prediction of pain outcomes in subgroups of patients with painful diabetic peripheral neuropathy

**DOI:** 10.1371/journal.pone.0207120

**Published:** 2018-12-06

**Authors:** Joe Alexander, Roger A. Edwards, Marina Brodsky, Luigi Manca, Roberto Grugni, Alberto Savoldelli, Gianluca Bonfanti, Birol Emir, Ed Whalen, Steve Watt, Bruce Parsons

**Affiliations:** 1 Pfizer Inc, New York, New York, United States of America; 2 Health Services Consulting Corporation, Boxborough, Massachusetts, United States of America; 3 Pfizer Inc, Groton, Connecticut, United States of America; 4 Fair Dynamics Consulting, Milan, Italy; University of Tokyo, JAPAN

## Abstract

Prior work applied hierarchical clustering, coarsened exact matching (CEM), time series regressions with lagged variables as inputs, and microsimulation to data from three randomized clinical trials (RCTs) and a large German observational study (OS) to predict pregabalin pain reduction outcomes for patients with painful diabetic peripheral neuropathy. Here, data were added from six RCTs to reduce covariate bias of the same OS and improve accuracy and/or increase the variety of patients for pain response prediction. Using hierarchical cluster analysis and CEM, a matched dataset was created from the OS (*N* = 2642) and nine total RCTs (*N* = 1320). Using a maximum likelihood method, we estimated weekly pain scores for pregabalin-treated patients for each cluster (matched dataset); the models were validated with RCT data that did *not* match with OS data. We predicted novel ‘virtual’ patient pain scores over time using simulations including instance-based machine learning techniques to assign novel patients to a cluster, then applying cluster-specific regressions to predict pain response trajectories. Six clusters were identified according to baseline variables (gender, age, insulin use, body mass index, depression history, pregabalin monotherapy, prior gabapentin, pain score, and pain-related sleep interference score). CEM yielded 1766 patients (matched dataset) having lower covariate imbalances. Regression models for pain performed well (adjusted R-squared 0.90–0.93; root mean square errors 0.41–0.48). Simulations showed positive predictive values for achieving >50% and >30% change-from-baseline pain score improvements (range 68.6–83.8% and 86.5–93.9%, respectively). Using more RCTs (nine vs. the earlier three) enabled matching of 46.7% more patients in the OS dataset, with substantially reduced global imbalance vs. not matching. This larger RCT pool covered 66.8% of possible patient characteristic combinations (vs. 25.0% with three original RCTs) and made prediction possible for a broader spectrum of patients.

**Trial Registration**: www.clinicaltrials.gov (as applicable): NCT00156078, NCT00159679, NCT00143156, NCT00553475.

## Introduction

Diabetes affects approximately 8.8% of adults worldwide [1], and around 50% of them develop polyneuropathy [2]. Moreover, 25% of those with diabetic neuropathy also develop neuropathic pain [3]. Reviews of current approaches to the treatment of painful diabetic peripheral neuropathy (pDPN) suggest that although progress is being made, there is still a pressing need to identify optimal ways to treat this condition [4]. Many efforts are under way to optimize therapy by segmenting patients on the basis of phenotypes, etiologies, and risk factors [5–9].

Promising approaches to treatment optimization may be identified more efficiently through integration of randomized controlled trial (RCT) data with non-randomized data [10]. Such integrated data hold the potential for delivering more useful evidence-based treatment-related information for clinicians [11], because randomized data focus on internal validity providing confidence about cause–effect relationships, and evidence from observational data focuses on external validity and provides confidence about relevance of a specific treatment choice. Integrating them quantitatively offers a way to reduce the covariate bias (one of the notable shortfalls of observational data) while still incorporating one of its core strengths related to external validity. Utilization of all available data in this manner holds the promise for optimizing therapy and reducing the impact of diabetic neuropathic pain globally.

Previously, we used data from three existing international RCTs and a large observational study (OS) in Germany [11] to assess treatment outcomes in pDPN patients treated with the α2δ ligand, pregabalin. Pregabalin is approved in Europe for the treatment of neuropathic pain [12], and in the United States for neuropathic pain associated with pDPN and spinal cord injury as well as for postherpetic neuralgia [13]. We implemented hierarchical clustering and applied coarsened exact matching (CEM) at the cluster level to match RCT patients with OS patients. Using these techniques, we were able to create patient subgroups whose pain outcomes could be effectively predicted with time series regressions with lagged variables as inputs at the subgroup level; however, the prior work had not optimized the regressions. The focus of the current analysis was to assess the clinical implications of applying such methods in a microsimulation platform with regressions optimized for prediction to deliver better care based on improved prediction of pregabalin treatment response for subgroups of patients. Our approach was based on applying methods that address the inherent time-varying nature of patient care and of the characteristics associated with their treatments for pDPN. Our goal was to determine whether the inclusion of data from six additional RCTs could further reduce, through CEM, the global imbalance (covariate bias) in the OS data. In addition, we evaluated whether the increase in available patient data could enable a more accurate prediction of treatment responses—first, in virtual patients who could be simulated, but ultimately, in a broader variety of actual patients. Finally, by utilizing machine learning techniques, we also sought to broaden the range of patients with pDPN for whom we could predict pain reduction outcomes with pregabalin.

## Methods

We utilized data from nine placebo-controlled RCTs designed to evaluate the efficacy of pregabalin for reducing pain scores in patients with pDPN (data on file for study A0081071; www.clinicaltrials.gov registration numbers: NCT00156078, NCT00159679, NCT00143156, and NCT00553475; the other trials were not registered on www.clinicaltrials.gov) [14–21]. The trials were conducted between March 1998 and March 2009 in Asia, Australia, Canada, Europe, Latin America, the Middle East, South Africa, and the United States. Patients received flexible- or fixed-dose pregabalin (75, 150, 300, or 600 mg/day) or placebo for 5–13 weeks (Table 1). Each of the nine studies shared fundamental inclusion criteria, including the requirement for patients to be aged ≥18 years; have a primary diagnosis of pDPN (type 1 or 2 diabetes mellitus with glycated hemoglobin (HbA1c) ≤11% and painful, distal, symmetrical, or sensorimotor polyneuropathy for ≥6 months); have an average pain score ≥4 [on an 11-point numeric rating scale (NRS), where 0 = *no pain* and 10 = *worst possible pain*] over a 7-day baseline period; and have a score ≥40 mm on the 0–100 mm visual analog scale of the Short-Form McGill Pain Questionnaire at screening and randomization [22]. Patients with creatinine clearance rates ranging from ≤30 to ≤60 mL/min were excluded, as were patients with any conditions that could jeopardize their health or confound assessment of pain due to pDPN. All studies were conducted in compliance with the ethics principles originating in or derived from the Declaration of Helsinki, internal review board requirements, or Good Clinical Practice guidelines, and all participants provided written informed consent before participation.

**Table 1:** Summary of patients from RCTs included in virtual Lab 2.0 by maintenance dose.

	5/6 Weeks Studies		12/13 Weeks Studies		Total RCT Patients	
Pregabalin dose	*n*	% of total	*n*	% of total	*n*	% of total
Flexible dose^a^	0	0.0	83	6.3	83	6.3
Flexible adjusted dose^b^	0	0.0	193	14.6	193	14.6
75 mg/day	59	4.5	0	0.0	59	4.5
150 mg/day	69	5.2	74	5.6	143	10.8
300 mg/day	124	9.4	297	22.5	421	31.9
600 mg/day	129	9.8	292	22.1	421	31.9
**Total**	**381**	**28.9**	**939**	**71.1**	**1320**	**100.0**

*n*, number of patients; RCT, randomized controlled trial.

^a^ Patients with 1–4 weeks escalation phase and 8–11 weeks maintenance (Protocol 1008–155).

^b^ Patients with 6 weeks escalation phase and 6 weeks maintenance (Protocol A0081030).

The primary efficacy measure in each study was change in pain score (on the 0–10 NRS) derived from entries in patients’ daily pain diaries (S1 Table). Pain responders at the 50% threshold were defined as those with [pain score at baseline–pain score (t)]/pain score at baseline ≥50% (where t = time at the end of the study). Secondary efficacy measures included change from baseline to end of study in pain-related sleep interference (PRSI) scores derived from daily sleep diaries in which patients rated how much their pain had interfered with their sleep using an 11-point NRS, where 0 = *pain does not interfere with sleep* and 10 = *pain completely interferes with sleep*.

The RCTs contained the following data for patients receiving active treatment: age, gender, body mass index (BMI), baseline pain score (0–10 NRS), baseline PRSI score (0–10 NRS), HbA1c normal or elevated, concomitant insulin use, pregabalin monotherapy, duration of diabetes, allodynia at baseline, average weekly pain (based on daily pain scores), average weekly PRSI (based on daily scores), prior gabapentin use, and past or current medical history of depression.

The OS data were from a 6-week, open-label study in standard outpatient settings in Germany [23]. The physicians were free to prescribe pregabalin 150–600 mg/day as either monotherapy or add-on therapy in accordance with the European Summary of Product Characteristics dosing schedule [12]. The OS collected the following data, which overlapped with data from the RCTs: age, gender, BMI, baseline pain score, baseline PRSI score, HbA1c (normal or elevated), ongoing insulin use (yes or no), prior gabapentin use, and past or current medical history of depression.

The OS was different from RCTs in the following aspects: it did not collect data on duration of diabetes and allodynia at baseline; it utilized flexible dosing of pregabalin; and it recorded additional information on duration of pDPN, history of sleep disorder, and history of anxiety. The OS recorded pain and PRSI scores at baseline and at weeks 1, 3, and 6 (in contrast to daily diary scores in the RCTs). Furthermore, only the OS dataset included ‘general feeling’ responses to three questions (*calm and relaxed*, *full of energy*, *sad and discouraged*) on a 6-point always-to-never scale, recorded at baseline and at weeks 1, 3, and 6. To impute missing data at weeks 2, 4, and 5, we used the EXPAND PROC (SAS Institute, Cary, North Carolina, USA) that utilizes second-order interpolation.

Another difference is that the six newly added RCTs were 12- or 13-weeks’ duration (S1 Table), whereas the original three RCTs were 6 weeks. For these six new RCTs, we utilized the 6-week outcomes for patients for the initial clustering, CEM, and time series regressions optimized using the least absolute shrinkage and selection operator (LASSO) method (described below). Then, during the simulation phase (described below), we incorporated data from the remaining weeks so as to take into account 50% pain responders at 6 weeks who were not responders at 12 or 13 weeks, as well as those who were not responders at 6 weeks but became responders by 12 or 13 weeks.

We sought to use the RCT data to reduce the level of bias in the distributions of covariates in the OS data. We used CEM to match the RCT data to the OS data [24] as we did in prior work [11].

We implemented hierarchical cluster analysis in the OS and then used CEM to match RCT patients to the patients in the OS clusters as described elsewhere [11], with one change. We used the Gower distance method rather than the Euclidean distance method because it could better represent mixed data types (eg, dichotomous, categorical, continuous) [25]. For each of the variables, we also analyzed whether the clusters in the matched dataset differed from one another in a statistically significant manner (as follows). For each pairwise comparison of variables with respect to the proportions of patients within a cluster, we applied Fisher’s exact test in three ways: (1) within the matched dataset used to derive the regressions with lagged variables; (2) within the validation dataset used for the initial evaluation of the regressions with lagged variables; and (3) between the clusters in the matched and validation datasets.

Next, we used the regression models to predict the behavior of a time series from past values, taking into account the existence of cross-correlations in the time series data to best represent multivariate analysis of the pain score at a given time lag in relation to:

1. Pain score at antecedent time lags;
2. PRSI score and other relevant time-dependent variables (eg, general feeling variables and prior treatment dose) at different time lags;
3. Specific patient demographic and/or medical history data likely to influence pain score.

We also used cross-correlation analyses to identify which variables to include in the regression models for each cluster. Candidate variables analyzed included: age cohort, gender, BMI, pDPN duration, past or current medical history of depression, previous use of gabapentin, history of pregabalin monotherapy, insulin use, general feeling (*full of energy*, *calm and relaxed*, *sad and discouraged*) at weeks 0, 1, 2, 3, 4, and 5; pain score at weeks 0, 1, 2, 3, 4, and 5; PRSI score at weeks 0, 1, 2, 3, 4, 5, and 6; and treatment dose at weeks 0, 1, 2, and 3. Other candidate variables included pain type (pain that was searing, throbbing, piercing, stifling, radiating, triggered by palpations, sharp, burning, cutting, twinging, pulsating, oppressive, or spasmodic), pain location (arms, legs, feet, trunk, or face), and pain frequency (intermittent or persistent).

The matched dataset was used to derive and calibrate the regressions for each of the clusters (the calibration or training dataset). Initially, we used a maximum likelihood method to estimate the parameter calibration of the regressions for each of the matched dataset clusters. We used forward and backward techniques to explore time lags and other variables to be included in each model for each cluster[26]. An initial validation of the regressions was implemented using patients in the OS *who did not match* with RCT patients and thus were not included in the calibration dataset. We plotted observed vs. predicted pain scores as well as the residuals to demonstrate confidence in the predictive validity of the regressions. A *t*-test of the time series of the observed vs. predicted pain scores was also performed in order to confirm that our data were consistent with the null hypothesis. Once we had achieved the best possible results with traditional techniques, we then employed a penalized regression method to improve the predictive capability of our regressions. We tested three methods for penalized regression: LASSO, adaptive LASSO, and elastic net. For LASSO selection, the penalty is placed on L1 norm of the regression coefficients; adaptive LASSO modifies the LASSO penalty by applying weights to each parameter that forms the LASSO constraint; and for elastic net, the penalty is on the combination of L1 and L2 norms of the regression coefficients [27]. Elastic net includes two tuning parameters, whereas LASSO and adaptive LASSO include only one. With our data, the three models performed very similarly for each of the six different clusters [See S2 Table].

We then decided to implement the simpler LASSO method given the similarities in the results of the three methods in this context to reduce the overall prediction error of the model based on the bias-variance tradeoff, which is achieved by decreasing the variance of the coefficient estimates while slightly increasing bias via shrinking the sum of absolute values of regression coefficients to zero [27, 28].

In order to analyze how we could predict pain scores over time, including the final score, for a novel patient, we used an agent-based modeling and simulation (ABMS) platform to create ‘virtual’ patients having different combinations of characteristics [29]. In order to define the domain of possible pain scores that a novel patient could face over the 6-week period, we adapted the Monte Carlo technique to perform 1000 virtual instances of a single novel patient. These virtual instances included fixed characteristics that were the same for each of the 1000 instances, along with time-varying characteristics that were different for each instance. We derived the probability densities for the time-varying components (eg, pain scores) in a three-step process of assigning a novel patient to a cluster, thereby triggering cluster-specific regression models to derive variations on patient responses, and then simulating a range of likely response trajectories (ie, the pain scores that a patient would most likely experience over time, starting from certain specific characteristics at baseline).

For the first step in the process, assigning a novel patient to a cluster, we utilized two instance-based machine learning techniques (k-Nearest Neighbor and Supervised Fuzzy C-Means). This cluster assignment then triggered which regression model would be applied to the simulation for the novel patient. Next, 1000 instances of the possible trajectories for the novel patient were created to reflect the possible trajectories of pain scores—effectively, 1000 virtual patients derived from the novel patient based on combinations of the fixed variables and the time-varying variables (described below). The regressions were applied weekly over the 6-week period. Dose was also assigned week by week considering the treatment given to the responder patients of the cluster achieving at least a 50% reduction in pain score.

The cluster-specific regressions included some parameters that were fixed (gender, age group, BMI, pDPN duration, taking insulin, pregabalin monotherapy, previous gabapentin, and past or current medical history of depression), general feeling variables at baseline (*full of energy*, *calm and relaxed*, *sad and discouraged*), and some that varied over time (PRSI scores in current and prior weeks, pain scores in prior weeks). For the variables with parameters that change during the 6 weeks’ treatment simulation, the ABMS platform relied on a method that would incorporate patients who were similar in how they changed over time. We utilized the k-Nearest Neighbor (kNN) approach for this task. The kNN approach defines ‘closest’ based on considering patients as vectors and calculating the Euclidean distance between the novel patient and the patients in the cluster, according to the considered variables. We adopted the convention of using the square root for ‘k’ [30]. In this situation, k becomes the square root of the cluster size. We then used the values in the trajectories from the ‘k’ closest patients to generate the Probability Density Functions (PDFs). These PDFs are then used as the source of the time-varying values (eg, PRSI scores and general feelings with a distinct PDF for each) for the 1000 instances of the novel patient. The variables were only used if they were significant for the regression model for a specific cluster to which the novel patient was assigned at the beginning. Once we obtained the k closest patients, we collected (1 week later: time t+1) their values for PRSI scores and general feeling variables, and plotted the probability densities for these k patients. For the subsequent week (time t+2), we performed the same steps, such that we were only considering the values of the time-varying variables at t+1 (and prior) for the ‘k’ patients who generated trajectories. This means that only those as close as possible to the novel patient would be simulated (ie, although all patients in the cluster were sorted by Euclidean distance, it was only the first ‘k’ patients who were considered for the PDF). The simulation returned the range of response trajectories that a patient would most likely experience over time, starting from specific characteristics at baseline. Distributions for pain score and responder status were displayed at the end of the 6-week simulation. The trajectories over the 6-week period were displayed in the Virtual Lab output in order to show how each virtual patient arrived at the final pain score at the conclusion of the simulation. We examined with simulation whether we could correctly predict the responder status of patients in the validation dataset using positive predictive value (PPV) and accuracy.

We also explored how we could extend analyses when we only used some of the available data [eg, no OS data beyond 6 weeks, no RCT data beyond 6 weeks (*N* = 3 studies), RCT data for 12–13 weeks (*N* = 6 studies)]. Hence, the Virtual Lab included a capability for estimating whether a candidate novel patient would maintain or modify beyond the end of week 6 his/her responder status achieved at the end of week 6. We accomplished extrapolation of responder status by focusing on the *monotonicity* of pain trajectories. Monotonicity is a measure of the extent to which a trajectory monotonically increases or decreases (S1 Fig) [31, 32]. In particular, we implemented the following steps:

1. Identification of two main outcome indicators: median pain score of the 1000 virtual patient trajectories at week 6 and the overall monotonicity of the cloud of those trajectories from week 0 to week 6;
2. Identification through a kNN approach of a subset of week 12–13 RCT patients who were closer to the simulated novel patient with respect to the two indicators noted in the prior step; and
3. Calculation of the monotonicity for patients in the six RCTs with 12–13 weeks of data based on the monotonicity of the kNN’s subset and use that information to predict patients who fell into three categories: patients who were responders at 6 weeks but became non-responders at 12–13 weeks; patients who were not responders at 6 weeks but became responders at 12–13 weeks; and patients who did not change their responder status.

## Results

**Fig 1:**
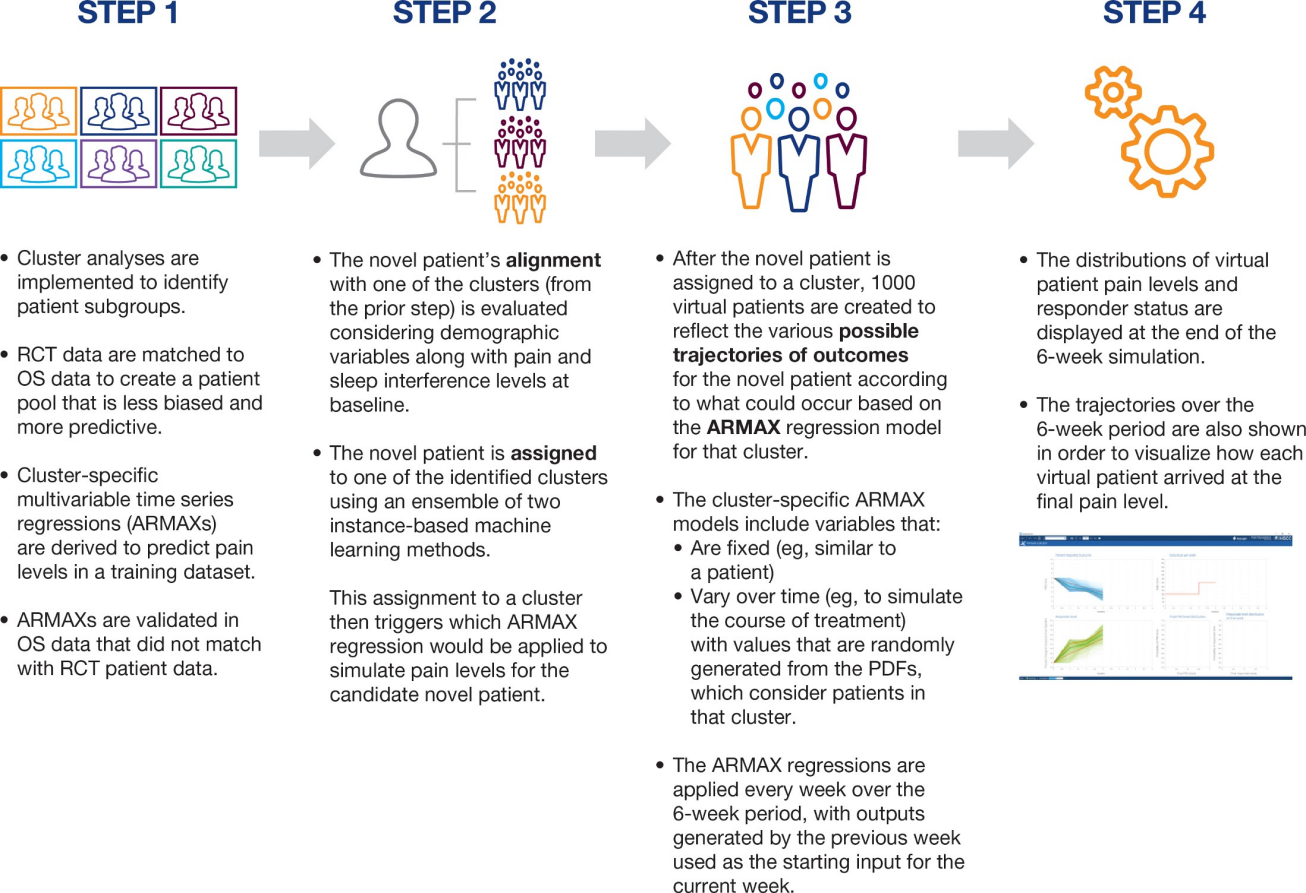
Simulation steps. *OS*, observational study; *PDF*, probability density function; *RCT*, randomized controlled trial.

The hierarchical cluster analysis of the OS (2642 patients) yielded six clusters (195–626 patients each) based on the semipartial *R*^*2*^ that measures the homogeneity of merged clusters [11] with the following clustering variables: gender, age, insulin use, BMI, past or current medical history of depression, pregabalin monotherapy, prior use of gabapentin, baseline pain score, and baseline PRSI score. The dendrogram from the cluster analysis, along with the dendrogram from the prior work, can be found in S2 Fig. Table 2 highlights the similarities and differences among the clusters within this dataset. After implementing the CEM, there were 1766 patients in the OS dataset (66.8%) who matched with 1077 patients from the RCTs for a total of 2843 patients in the matched dataset. These 1077 RCT patients represented 81.6% of the 1320 total RCT patients in the nine RCTs. Of these matched RCT patients, 19.9% matched to one cluster, 19.6% to two clusters, and 60.5% to three or more clusters. The reduction in the imbalance scores for the clusters after adding in the RCT patients (ranging from 46.6% to 56.7% depending on the cluster) suggests that the process as shown in step 1 of Fig 1 notably reduced the bias of covariates in all six clusters (S3 Table). The six additional RCTs enabled an additional 562 patients (46.7%) in the OS dataset to match; the matched dataset of 2843 patients was 86.1% higher (1315 additional patients) than the prior work with only three RCTs.

**Table 2:** Baseline patient characteristics from calibration dataset (*N* = 1766), by cluster.

	1	2	3	4	5	6	Total
*n*	431	189	437	266	127	316	1766
Females (%)	0.0	47.1	38.0	100.0	26.8	41.8	38.9
Age (years), mean (SD)	60.2 (9.3)	62.9 (8.6)	62.9 (8.5)	62.2 (8.5)	61.3 (9.7)	63.7 (8.3)	62.2 (8.9)
Age group (years), %							
0–44	4.8	0.5	0.9	1.9	4.7	1.3	2.3
45–64	63.6	54.5	53.3	59.8	60.6	53.5	57.5
65–74	27.4	37.1	38.4	32.7	26.8	37.3	33.7
75+	4.2	7.9	7.3	5.6	7.9	7.9	6.5
BMI (kg/m^2^)							
Mean (SD)	28.1 (3.5)	29.5 (4.5)	29.7 (4.6)	28.9 (4.6)	28.5 (4.4)	28.4 (3.8)	28.8 (4.2)
Normal (%)	13.5	13.2	10.8	17.7	13.4	14.8	13.6
Overweight (%)	64.5	47.1	49.2	47.7	59.1	58.2	54.8
Obese (%)	22.0	39.7	40.0	34.6	27.6	27.0	31.5
Baseline pain score							
Mean (SD)	6.3 (1.3)	7.1 (1.3)	6.6 (1.4)	6.4 (1.3)	6.3 (1.4)	6.3 (1.3)	6.5 (1.4)
Pain severity category (%)							
Mild (0–3)	-	-	-	-	-	-	-
Moderate (4–6)	55.4	31.7	46.0	50.0	54.3	56.3	49.8
Severe (7–10)	44.6	68.3	54.0	50.0	45.7	43.7	50.2
Baseline PRSI score							
Mean (SD)	5.3 (2.2)	6.8 (1.9)	5.9 (2.2)	5.8 (1.9)	5.6 (2.1)	5.5 (2.0)	5.7 (2.1)
PRSI category							
Mild (0–3)	21.1	6.9	13.9	12.1	20.5	15.5	15.4
Moderate (4–6)	43.4	30.2	41.7	50.0	44.9	51.6	44.1
Severe (7–10)	35.5	62.9	44.4	37.9	34.6	32.9	40.5
Duration of pDPN (years), %							
0 to ≤5	28.8	15.9	21.3	25.9	24.4	24.4	24.0
>5 to ≤10	23.2	21.7	23.6	21.8	24.4	22.2	22.8
>10 to ≤15	25.5	24.9	24.0	27.1	22.8	18.3	23.8
>15 to ≤20	11.1	15.3	12.8	15.4	10.2	18.7	13.9
>20 to ≤25	3.7	5.8	5.5	2.6	7.1	6.0	4.9
>25	7.7	16.4	12.8	7.1	11.0	10.4	10.5
Past or current medical history of depression (%)	0.0	100.0	2.1	0.0	2.4	0.0	11.4
Prior or current therapy (%)							
Pregabalin monotherapy	100.0	34.9	59.9	100.0	64.6	0.0	62.7
Gabapentin	0.0	11.1	2.8	0.0	100.0	1.0	9.2
Insulin	0.0	34.9	99.8	1.5	48.8	7.9	33.6
Full of energy at baseline (%)							
Always	1.2	1.1	0.7	1.1	0.0	0.6	0.8
Mostly	7.7	1.1	5.3	3.4	3.9	4.4	4.9
Fairly often	13.5	2.1	7.1	10.5	13.4	7.9	9.2
Sometimes	29.9	17.5	29.5	31.2	25.9	20.9	26.8
Seldom	40.6	49.7	43.3	48.1	43.3	57.9	46.7
Never	7.2	28.6	14.2	5.6	13.4	8.2	11.6
Calm and relaxed at baseline (%)							
Always	2.1	2.1	2.9	0.8	0.8	1.3	1.9
Mostly	15.3	4.2	11.2	13.2	12.6	13.3	12.2
Fairly often	16.9	9.0	16.5	15.8	20.5	13.6	15.5
Sometimes	32.2	20.1	29.9	28.9	29.9	31.9	29.7
Seldom	31.8	47.6	33.9	38.4	29.1	36.7	35.7
Never	1.6	16.9	5.5	3.0	7.1	3.2	5.1
Sad and discouraged at baseline (%)							
Always	1.6	6.4	2.9	1.9	3.9	1.6	2.7
Mostly	15.3	35.9	17.6	15.4	15.8	15.5	18.2
Fairly often	28.3	33.3	27.0	26.3	26.8	31.0	28.6
Sometimes	29.3	15.3	29.5	30.8	26.8	33.2	28.6
Seldom	20.2	8.5	16.3	21.8	22.1	15.5	17.5
Never	5.3	0.5	6.6	3.8	4.7	3.2	4.5
Pain responders at 50% threshold at endpoint (%)	87.2	72.5	79.6	78.6	77.9	79.4	80.4
Daily treatment dose (mg)							
75	3.5	3.7	4.8	4.5	2.4	6.0	4.4
150	38.3	31.2	35.7	38.7	31.5	34.2	35.7
300	53.8	59.8	54.7	52.6	61.4	55.4	55.3
600	4.4	5.3	4.8	4.1	4.7	4.4	4.4
Other	0.0	0.0	0.0	0.0	0.0	0.0	0.0

BMI, body mass index; pDPN, painful diabetic peripheral neuropathy; PRSI, pain-related sleep interference; RCT, randomized controlled trial; SD, standard deviation.

The OS covered 69.3% of the 192 possible combinations of the following characteristics: gender, age group (≤44, 45–64, 65–74, ≥75 years), BMI group (normal, overweight, obese), insulin use, prior gabapentin use, and pain categories at baseline divided into moderate (4–6) or severe (7–10). The nine RCTs and the combined dataset covered 66.1% and 76.6%, respectively, of the 192 possible combinations of these patient characteristics.

The Fisher’s exact test results (Table 3) for the proportion of patients within a cluster for each pairwise comparison of the variables showed that at least two thirds of the pairwise comparisons were statistically different for:

1. Matched dataset used for regression model calibration: gender, insulin use, past or current medical history of depression, prior gabapentin use, and pregabalin monotherapy;
2. Validation dataset used for initial regression model evaluation: insulin use, prior gabapentin use, and pregabalin monotherapy; and
3. Matched vs. Unmatched datasets: gender, age group, BMI group, insulin use, PRSI score at baseline, and pregabalin monotherapy.

**Table 3:** Statistical comparison of clusters within the matched dataset, within the validation dataset, and between the matched and validation datasets^a^.

	Across Clusters Within Calibration Dataset (# of Unique Clusters out of 15 Pairwise Comparisons)	Across Clusters Within Validation Dataset (# of Unique Clusters out of 15 Pairwise Comparisons)	Calibration vs. Validation Dataset (# of Unique Clusters out of 36 Pairwise Comparisons)
Gender	13 of 15 (87%)	9 of 15 (60%)	30 of 36 (83%)
Age group	6 of 15 (40%)	5 of 15 (33%)	36 of 36 (100%)
BMI	8 of 15 (53%)	6 of 15 (40%)	29 of 36 (81%)
Insulin use	15 of 15 (100%)	13 of 15 (87%)	27 of 36 (75%)
Past or current medical history of depression	11 of 15 (73%)	6 of 15 (40%)	13 of 36 (36%)
Prior gabapentin	11 of 15 (73%)	10 of 15 (67%)	23 of 36 (64%)
Pregabalin monotherapy	13 of 15 (87%)	13 of 15 (87%)	26 of 36 (72%)
PRSI score at baseline	9 of 15 (60%)	5 of 15 (33%)	36 of 36 (100%)
Pain score at baseline	7 of 15 (47%)	2 of 15 (13%)	14 of 36 (39%)
Dose	0 of 15 (0%)	0 of 15 (0%)	2 of 36 (6%)

BMI, body mass index; PRSI, pain-related sleep interference.

^a^ Each pairwise comparison of one variable in one cluster to one variable in another cluster was evaluated using Fisher’s exact test. The number of significant *P*-values at *P* < 0.05 were tallied, and those counts are shown in the table.

All of the final regression models that estimated weekly pain scores for the matched data performed well after implementing the regularization with LASSO, with adjusted *R*^*2*^ ranging from 0.97 to 0.98, and root mean square errors ranging from 0.47 to 0.51 (Table 4). The most influential variables were those associated with time-lagged relationships. The regressions before regularization with LASSO also performed well but with many more variables (See S4 Table).

**Table 4:** Regression model Input variables and resulting regression coefficients by cluster for the calibration dataset.

**Regression Model Input Variables**	**Final Output Regression Coefficients, by Cluster^a^**					
	**1**	**2**	**3**	**4**	**5**	**6**
y-intercepts for regression models, not variables	-	-	-	-	-	-
Age cohort (75+)^f^ (**x5**)	-	0.1039	-	-	-	-
General feeling: calm and relaxed (t = 0)^e^ (**x6**)	0.0354	0.0557	-	0.0671	0.0150	0.0107
General feeling: full of energy (t = 0)^e^ (**x7**)	-	-	0.0162	0.0043	0.0133	0.0449
Pain score (t-1)^b^ (**x1**)	0.6229	0.7590	0.7605	0.6294	0.6415	0.6652
PRSI score (t)^d^ (**x2)**	0.2461	0.1417	0.2393	0.2146	0.2234	0.1945
PRSI score (t-3)^c^ (x**3**)	-	-0.0455	-0.0707	-	-	-
Dose (t-3)^c^ (**x4**)	0.0002	0.0002	0.0002	0.0003	-	-
Model performance measures applied	Performance, by cluster					
	1	2	3	4	5	6
Likelihood ratio *P*-value	< 0.0001	< 0.0001	< 0.0001	< 0.0001	< 0.0001	< 0.0001
*Adjusted R*^*2*^	0.97	0.98	0.98	0.98	0.97	0.97
Root mean square error	0.49	0.51	0.49	0.47	0.51	0.50
Observed vs. estimated responders (Student’s *t* test *P-*value)^g^	0.55	0.64	0.46	0.90	0.33	0.83

PRSI, pain-related sleep interference.

^a^ The first number in each column is the regression intercept value. Blank spaces in columns indicate that the associated row variable was not a predictor in the final model for that cluster.

^b^ (t-1) indicates 1 week before prediction.

^c^ (t-3) indicates 2 weeks before prediction.

^d^ (t) indicates the same week of the prediction.

^e^ (t = 0) indicates baseline.

^f^ Dummy variables have been introduced for categorical variables. For example, Age cohort (75+) is the dummy variable related to the “75+” value of the Age cohort variable; it means that the corresponding coefficient affects only patients having Age cohort = 75+, but not patients with different values of the Age cohort variable (i.e. 0–44, 45–64, and 65–74).

The regression model inputs were assigned unique variable names, x1–x7, and are represented in the cluster-specific regression equations below:

**Equations for the regression models** (where ‘y’ is the fitted pain score) both for H.1

**CLUSTER 1:** y = 0.6229**x1** + 0.2461**x2** + 0.0002**x4** + 0.0354**x6**

**CLUSTER 2:** y = 0.7590**x1** + 0.1417**x2**–0.0455**x3** + 0.0002**x4** + 0.1039**x5** + 0.0557**x6**

**CLUSTER 3:** y = 0.7605**x1** + 0.2393**x2**–0.0707**x3** + 0.0002**x4** + 0.0162**x7**

**CLUSTER 4:** y = 0.6294**x1** + 0.2146**x2** + 0.0003**x4** + 0.0671**x6** + 0.0043**x7**

**CLUSTER 5:** y = 0.6415**x1** + 0.2234**x2** + 0.0150**x6** + 0.0133**x7**

**CLUSTER 6:** y = 0.6652**x1** + 0.1945**x2** + 0.0107**x6** + 0.0449**x7**

^g^ The regressions estimate pain score, but we also want to be able to identify whether that patient is a responder at different thresholds (eg, 50% or 30% reduction in pain score). Hence, we wanted to confirm estimation of responder level based on the regression for pain score.

The following were influential in several cluster-specific regressions: pain in prior weeks, PRSI, treatment dose, *feeling calm and relaxed*, and *feeling full of energy*. Age group was influential in one cluster-specific regression.

**Fig 2:**
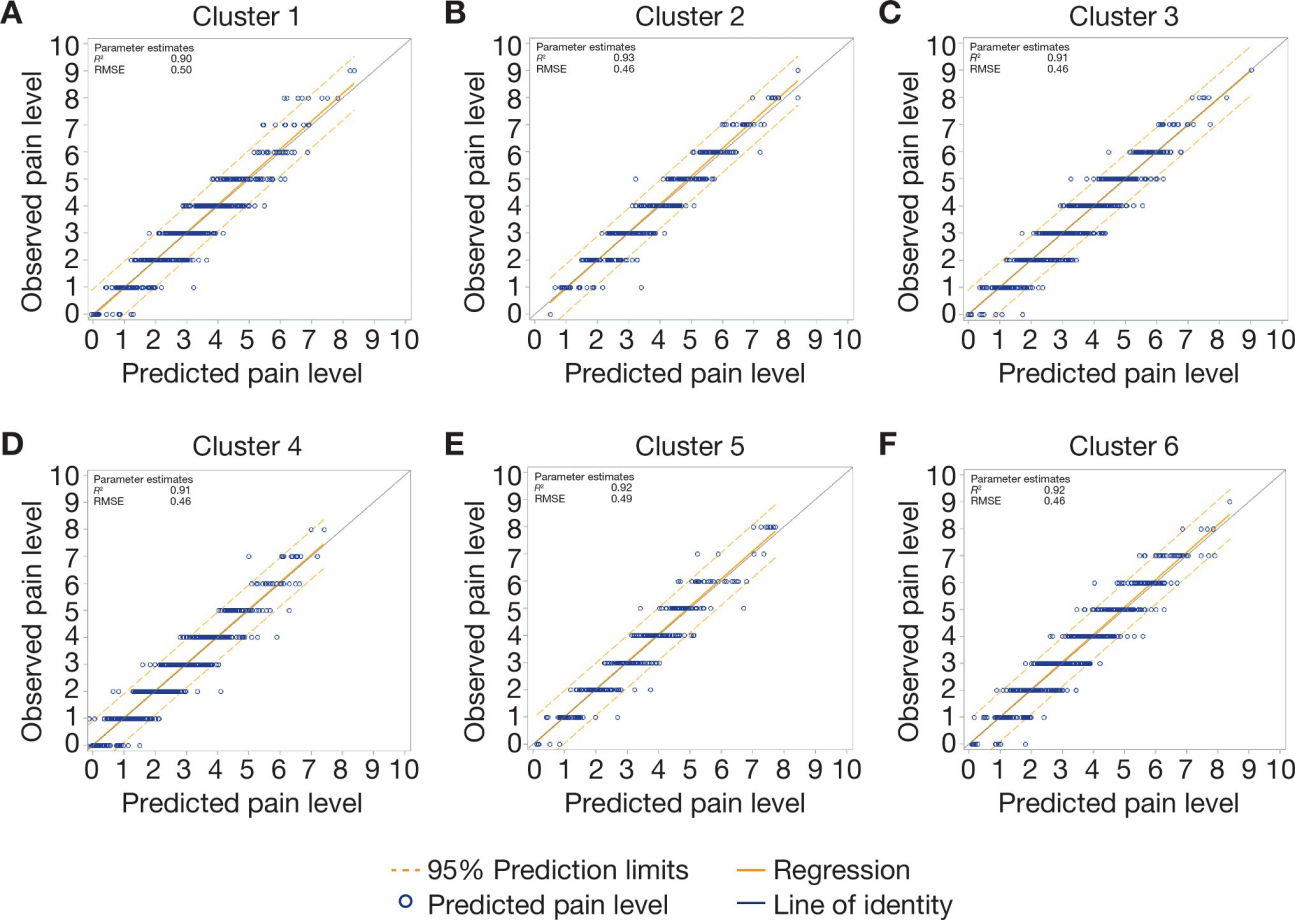
Plots of observed vs. predicted pain scores and residuals in validation dataset. *RMSE*, root mean square error.

We also evaluated how well these regression models predicted responders in the OS patients who did *not* match with RCT patients (validation dataset *N* = 876). When comparing observed pain scores in the validation dataset with those predicted using the regression models derived from the calibration dataset, two-sample *t*-tests found statistical similarity for all clusters (all *P* > 0.05). The regressions of the scatterplots of observed vs. predicted pain scores showed *R*^*2*^ values of 0.97, 0.98, 0.98, 0.98, 0.98, and 0.97 for clusters 1–6, respectively (see Fig 2 for plots of observed vs. predicted pain scores and the residuals for these plots for each cluster).

Table 5 shows the PPV and accuracy results for virtual patients achieving a 50% reduction in pain scores compared with actual patients. Overall, the PPV was 77.8% (ranging from 68.6% to 83.8% for an individual cluster). If we used the 30% pain responder definition, the PPV and accuracy improved to 91.6% (ranging from 86.5% to 93.9%, depending on the cluster) and 91.6% (ranging from 86.5% to 93.9%), respectively.

**Table 5:** PPV and accuracy for best scenario.

Model Performance Measures Applied	Performance, by Cluster						
	1	2	3	4	5	6	Overall
PPV at 30% pain responder level threshold	93.9%	86.5%	92.5%	93.4%	89.6%	90.0%	91.6%
Accuracy at 30% pain responder level threshold	93.9%	86.5%	92.5%	93.4%	89.6%	90.0%	91.6%
PPV at 50% pain responder level threshold	81.5%	68.6%	78.0%	83.8%	68.7%	75.9%	77.8%
Accuracy at 50% pain responder level threshold	81.8%	64.6%	75.9%	82.7%	68.7%	75.3%	76.5%

PPV, positive predictive value.

**Fig 3:**
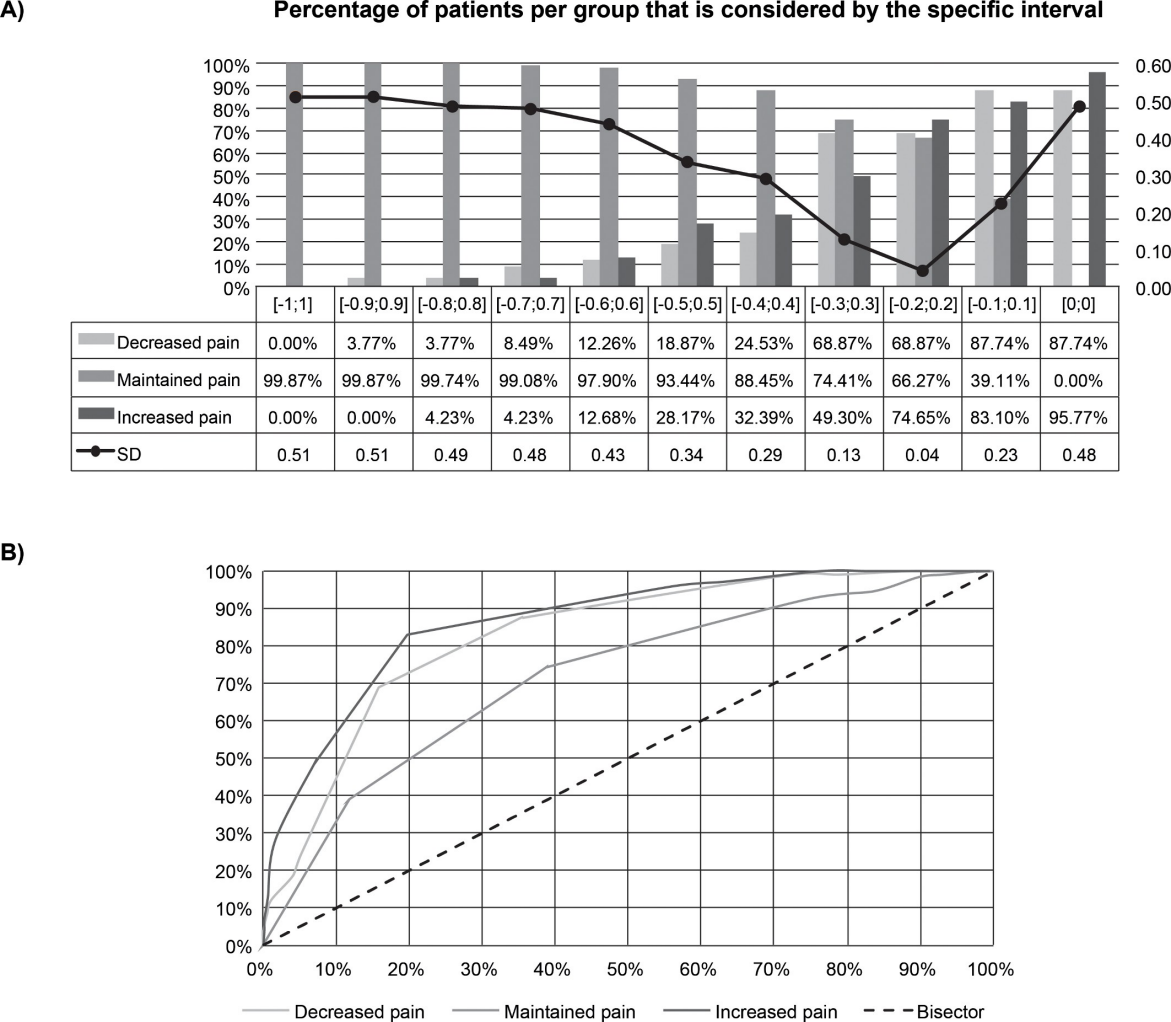
A) Monotonicity results. *N* = 106 for decreased pain, *N* = 762 for maintained pain response from weeks 6 to 12–13 (358 responders + 404 non-responders), *N* = 71 for increased pain. B) ROC curves for monotonicity prediction of pain beyond 6 weeks. Correct prediction based on majority of simulated patient outcomes.

Results for the monotonicity analysis were based on the following patient groups: (a) increased pain: 7.6% of patients were responders at week 6 and became non-responders at week 12 or 13 (*N* = 71); (b) decreased pain: 11.3% of patients were not responders at week 6 but became responders by week 12 or 13 (*N* = 106); and (c) maintained pain response from weeks 6 to 12–13: 81.2% of patients did not change their responder status between weeks 6 and week 12 or 13 (*N* = 358 for responders and *N* = 404 for non-responders) (S5 Table). We looked at the monotonicity in each of these three groups and identified monotonicity categories: monotonicity above a positive threshold (x>0.2), monotonicity between positive and negative thresholds (–0.2≤x≤0.2), and monotonicity below a negative threshold (x<–0.2). The thresholds of –0.2 and +0.2 produced balanced combinations in these groups with the lowest standard deviation of the mean monotonicity for the group. Results in Fig 3A show these thresholds leading to the more balanced combination of the three patient groups [ie, decreased pain (*n* = 106), maintained pain response from weeks 6 to 12–13 (*n* = 358+404 = 762), increased pain (*n* = 71)] and the lowest standard deviation of the mean monotonicity.

The accuracy for how well we could correctly predict whether a patient would further decrease his/her pain score, maintain his/her pain score, or increase his/her pain from 6 to 12–13 weeks was 82.4%, 71.9%, and 89.5%, respectively. Fig 3B shows the receiver operating characteristic (ROC) curves for these three groups.

## Discussion

These results demonstrate the potential value of integrating patient data from RCT and OS sources and developing subgroup-specific regressions for prediction of therapeutic response (discussed further below). The inclusion of additional RCTs in this analysis enabled a 46.7% increase in matched OS patients (from 1204 to 1766) compared with earlier work. A high degree of imbalance often occurs in OSs because their assignments to treatments are not random. A reduction of this imbalance can be achieved, however, through a matching of OS patients with those from relevant RCTs in which the covariates are, in principle, more highly balanced owing to the randomized design. Matching is thus intended to identify a better balance in the multidimensional distribution of covariates, resulting in the lower covariate bias desired to establish better explanatory models of potential causal relationships among measured variables [33]. Global imbalance after matching was also substantially reduced in all six clusters (with the nine RCTs) instead of in only five of them (with three RCTs), with all of the resulting clusters having less covariate bias and being able to support robust prediction. Although there were only small gains in terms of prediction, the larger RCT pool now allowed prediction for a broader spectrum of patients because the nine RCTs covered 66.8% of possible combinations of patient characteristics compared with 25.0% with the three original RCTs. That capability for predicting the response to treatment in a wider range of patient types was further extended owing to the ensemble machine learning techniques that relaxed the requirements for matching a novel patient to a cluster; response trajectories of patients matched with a broader combination of characteristics could be modeled, simulated, and ultimately predicted.

Consistent with prior work, the heterogeneity of patients with pDPN was confirmed by the existence, in the OS, of six clusters of patients defined by different patterns of patient variables (Table 2). Most baseline variables did not sort into a single cluster (age group, BMI group, duration of pDPN, pain score, PRSI score, feeling *full of energy*, *calm and relaxed*, *sad and discouraged*), but rather varied in their combinations. A few were present in isolation (eg, all females in one cluster and all males in one cluster, no current use of insulin in one cluster, pregabalin monotherapy in two clusters but completely absent in one cluster, prior use of gabapentin in one cluster but completely absent in two clusters, past or current medical history of depression in one cluster but completely absent in three clusters), but they were found in various proportions in the remaining clusters. These findings demonstrate the substantial level of complexity and interaction among variables and underscore the challenge facing clinicians who are selecting appropriate treatment and dose for individual patients. The need to process enormous amounts of disparate clinical information (in the absence of effective modeling tools) may explain why clinicians often experience a great degree of uncertainty, but might also depend upon intuition rather than on a clearly defined rationale about which patients are likely to respond better to certain therapeutic approaches. For example, one could assume that the existence of females in one cluster suggests that a subgroup of females might be similar; however, most of the females are spread across five subgroups. Thus, the trait of being female combined with other characteristics is what defines their placement within a specific cluster.

There were 50% pain responders and non-responders in every cluster, and all treatment doses were present in all clusters. The clusters were quite similar to the clusters from the prior work; however, there were fewer instances of clusters with only one state for the dichotomous variables. This finding might be due to the change from using Euclidean distance to Gower distance, with its ability to better identify distances with different types of variables.

The pairwise comparison of variables in the six clusters (Table 3) supported prior work related to the distinctive *patterns* of variables in each cluster. Some overlap was found in pairwise comparisons for some clusters within the matched OS patient dataset, within the unmatched OS patient dataset (validation dataset), and between the two datasets. As with the prior work, dose was not influential in defining a cluster; however, based on its presence in the majority of the cluster-specific regression equations, dose plays an important role in predicting whether a patient is likely to respond to pregabalin therapy. Since some patients require titration above a 300 mg dose and others do not [12, 13}, it is not surprising that titration was not predictive in all of the cluster-specific regressions.

Fixed patient variables combined with ‘on-treatment’ variables in different ways to predict responses in the different clusters, as seen in the regression results in Table 4. The parameters in the regression models before regularization (See S4 Table) reinforced the reciprocal influences between pain and PRSI [34] and dose in prior weeks [35, 36]. They also showed the relevance of selected psychosocial variables for certain subgroups of patients, but not others, as has been shown in other studies [37, 38]. Other variables such as age and insulin use were the only significant predictors in one of the cluster-specific regression models before we optimized them using the LASSO method, although demographic and other medical care characteristics are often used as a basis for subgroup analyses in clinical studies of pain [7, 37, 39–42]. The optimized regressions for prediction, however, had fewer variables suggesting that when prediction is the goal, specific regression regularization techniques are warranted. The robust adjusted *R*^*2*^ and root mean square error results for the regressions (Table 4) support the strong predictive capability afforded by using regression models with lagged variables as inputs for predicting the magnitude of response to pregabalin when useful subgroups (clusters) of patients are created first, as was found for prior work [11]. By using regressions that take into account past values, we were better able to evaluate the possible importance of other variables for the purposes of prediction (as opposed to description).

The regressions predicted pain responses even in OS patients who had not matched, thus providing significant validation for these regressions that were generated from the entirely different matched dataset. Plots of observed vs. predicted pain scores by cluster shown in Fig 2 validate the good predictive performance of the regressions. Two-sample *t*-test results corroborate these findings (S6 Table).

**Fig 4:**
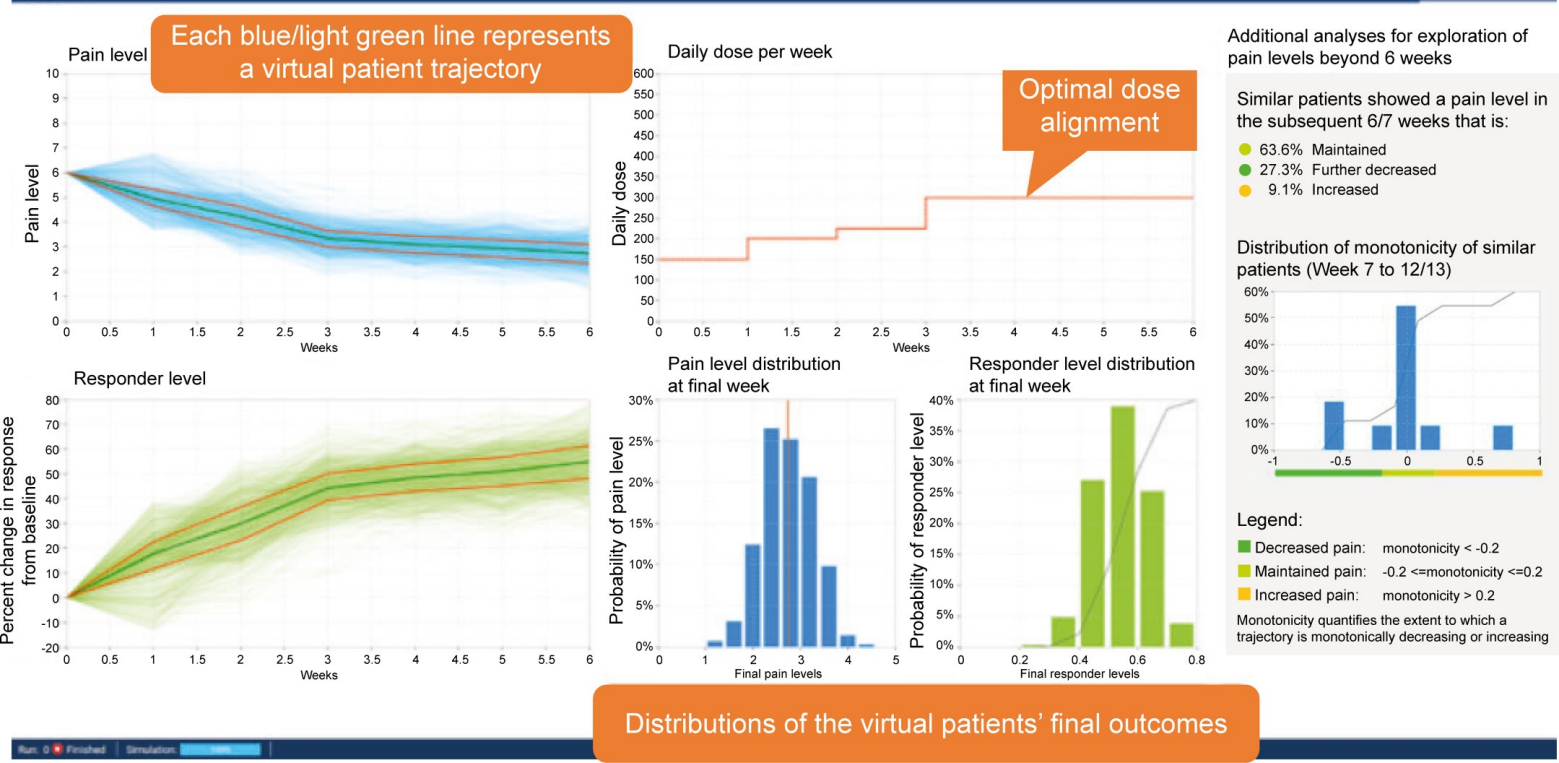
Simulation output.

The cluster-specific regressions are the basis for the Virtual Lab’s performance in predicting a novel patient’s treatment outcomes because they establish and provide for not only weekly pain scores, but also weekly patient variations of response through incorporation of ‘on-treatment’ variables as well as fixed ones. Fig 1 provides an overview of the various steps in the prediction process. Fig 4 shows an example of the output from the simulation steps described in Fig 1. Hence, proper assignment of a novel patient to a specific cluster is very important given the role of the regressions in the platform.

Consequently, we focused substantial efforts in this next generation of the platform on how to broaden the characteristics of patients who could be assigned to a cluster—as well as to represent the uncertainty in the predicted outcomes accordingly, while maintaining or enhancing the accuracy of the prediction.

The PPV results (Table 5) indicated that 68.6–83.8% of the time (depending on the cluster) and 77.8% overall, we correctly predicted patients who attain the 50% reduction from baseline in pain scores. Correct predictions (PPV and accuracy) improved to 91.6% overall with a range of 86.5–93.9% at the 30% threshold for pain score reduction. From a clinical decision-making point of view, many clinicians and patients would agree that a 30% reduction in pain is a clinically meaningful therapeutic response [43].

We also explored a way to introduce information about outcomes that could occur after 6 weeks of treatment by utilizing data from longer RCTs (12 or 13 weeks) even though we lacked OS data for this time period. Overall, 81.2% of the patients who had or had not achieved a 50% pain reduction at 6 weeks maintained that outcome at 12 or 13 weeks (S6 Table). Thus, we estimated—using our monotonicity thresholds—the likelihood for a novel patient after 6 weeks of treatment improving his/her response to reach the 50% threshold in pain score reduction (11.3%) or for falling below that threshold (7.6%). We discovered that individuals with similar monotonicity were showing similar evolutions in pain scores after week 6 (falling into the categories of remaining at the same pain score or increasing or decreasing). This estimation was possibly based on identifying similar patients in the cluster rather than on relying upon global average rates for these phenomena. We incorporated this extension of prediction capability into the Virtual Lab by calculating the overall monotonicity of the aggregation of 1000 possible pain trajectories for the simulated novel patient. We also calculated the final pain score of the simulated novel patient as the median of the 1000 simulated final pain scores. We then identified the patients in the expanded RCT dataset that are closer to the simulated novel patient (using the kNN approach) on the basis of monotonicity at week 6 and pain at week 6. We used these nearest neighbors to calculate the monotonicity from week 6 to 12/13 in order to identify to which category the novel patient could be assigned (decreased, maintained, or increased). The results of how many of the RCT nearest neighbor patients fell into each category are plotted in a histogram. S1 Fig shows an example of the histogram and how the monotonicity analyses could be incorporated into the Virtual Lab output to determine whether a novel patient would or would not maintain pain score in the subsequent 6–7 weeks.

These results demonstrate the potential utility of integrating patient data from RCT and OS sources to identify patient subpopulations that will manifest different degrees of therapeutic response to a particular treatment. We have shown how a combination of carefully selected modeling and microsimulation approaches can be used to predict which patients with pDPN may have a higher probability of responding to pregabalin and at which dose. These findings also highlight the importance of tracking changes over time in certain variables (eg, pain and PRSI) after the initiation of treatment in order to adjust baseline predictions accordingly. The use of ABMS provided a mechanism for introducing patient variability into the analyses by integrating the on-treatment variables that changed over time with fixed patient characteristics and simulating the resulting on-treatment pain response. Future research can explore the range of potential applications for this approach of clustering, matching, and time series regressions with lagged variables as inputs.

### Limitations and future work

A limitation of our analyses is that for all studies included, we used data only from those subjects who completed the studies, so more work would be needed to determine if the results are generalizable to other patient populations (eg, patients who discontinued the studies owing to adverse events). However, prior studies that occurred over a decade have found that treatment-emergent adverse events in pregabalin-treated patients infrequently lead to discontinuation [39] and that the most common side effects of pregabalin resolve over time [44]. Given that we are including lagged variables as inputs, events not reflected in the variable included could affect the time series data. Using traditional regression techniques with added variables may be correlated (e.g., high variability, correlated predictor variables), which is a limitation that motivated us to add a penalized regression method (LASSO) to regularize the predictive capability of the cluster-specific regressions.

Subsequent Virtual Lab work will focus on predicting discontinuations and adverse events using these same approaches, along with refining how we predict outcomes beyond 6 weeks. Also, we have not yet tried prospectively to predict actual new patient outcomes using our platform. Future work will need to examine how such efforts could work in actual practice. We need to explore how the proposed approach, including a capability for dynamic real-time updates, could be used in providing care to patients. Finally, these findings are specific to patients with pDPN, and not all patient variables associated with pDPN have yet been studied. Other clinical circumstances may require less or more complex approaches to enable prediction.

## Conclusions

The inclusion of data from six additional RCTs expanded the combinations of patient characteristics and outcomes that could be predicted using our methodological approach (consisting primarily of hierarchical cluster analysis, CEM, regressions optimized based on use of shrinking and penalty search algorithms, and microsimulation). In addition, matching of substantially more RCT patients to OS patients strengthened the representativeness of OS patients while maintaining strong overall predictive power. These analyses reinforced the predictive value of utilizing patient subgroups that reflect more complex patterns of fixed patient characteristics and ‘on-treatment’ variables that change over time.

## Supporting information

S1 Table Summary of patients from RCTs included in virtual Lab 2.0, by pregabalin dose (Table 1 with Supplemental Notes). (DOCX)

S2 Table Comparison of LASSO, adaptive LASSO, and elastic net regressions. (DOCX)

S3 Table CEM results. (DOCX)

S4 Table Regression model input variables and resulting regression coefficients by cluster for the calibration dataset Before Regularization. (DOCX)

S5 Table Change in responder status at 6 weeks compared with the end of the study. (DOCX)

S6 Table Regression model performance by cluster: Two-sample t-Testa. (DOCX)

S1 Fig Supplemental information on monotonicity of pain trajectory. (TIF)

S2 Fig Dendrograms of the clusters. (A) Virtual Lab 2.0. (B) Virtual Lab 1.0.
(TIF)
