# Correction: Using time series analysis approaches for improved prediction of pain outcomes in subgroups of patients with painful diabetic peripheral neuropathy

**DOI:** 10.1371/journal.pone.0212959

**Published:** 2019-02-26

**Authors:** Joe Alexander, Roger A. Edwards, Marina Brodsky, Luigi Manca, Roberto Grugni, Alberto Savoldelli, Gianluca Bonfanti, Birol Emir, Ed Whalen, Steve Watt, Bruce Parsons

Fig 1 is incorrect. The authors have provided a corrected version here.

**Fig 1:**
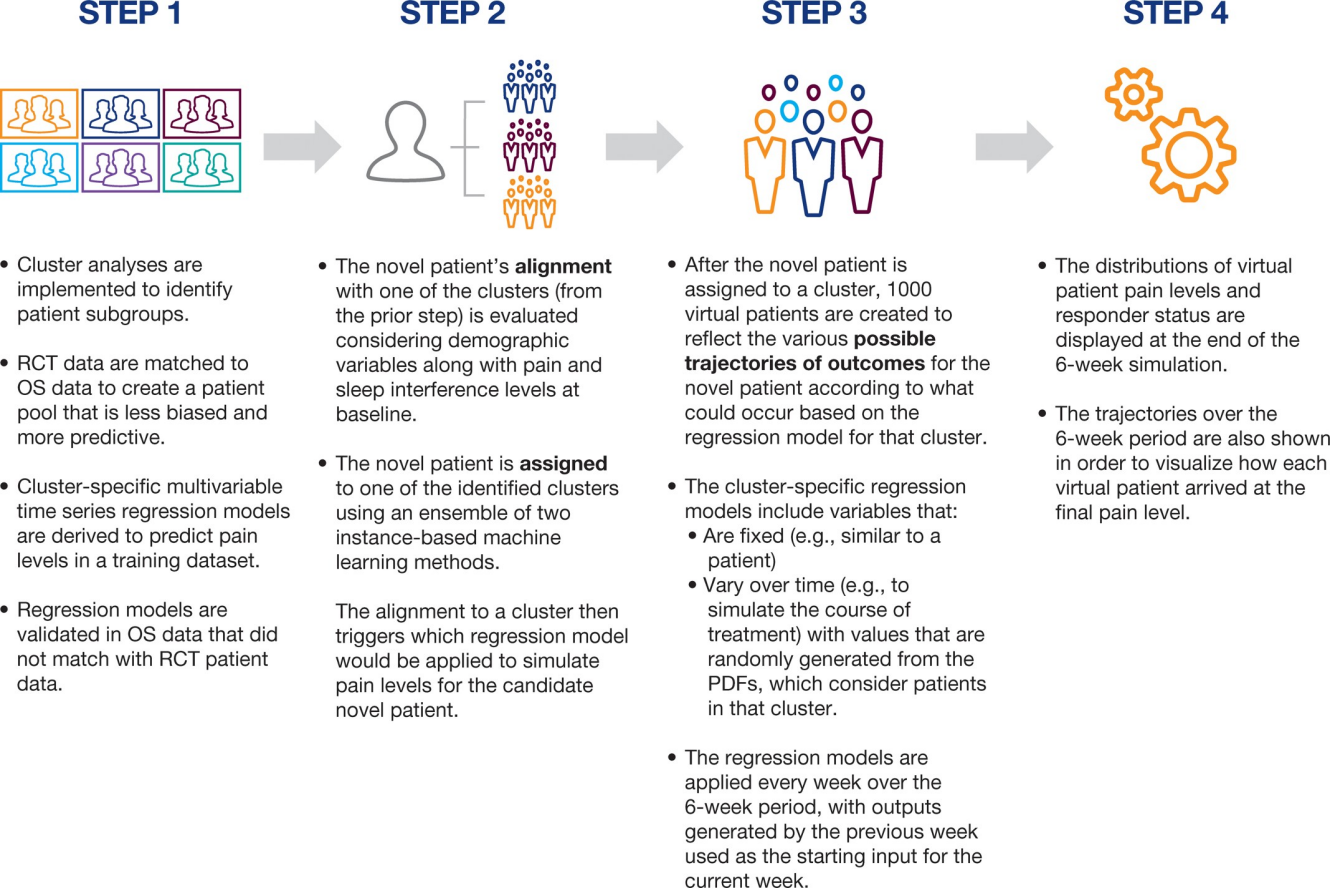
Simulation steps. *OS*, observational study; *PDF*, probability density function; *RCT*, randomized controlled trial.
