# Supplementary material for: Using time series analysis approaches for improved prediction of pain outcomes in subgroups of patients with painful diabetic peripheral neuropathy
Source: PLoS One. 2018 Dec 6;13(12):e0207120. doi: 10.1371/journal.pone.0207120 (PMC6283469; doi:10.1371/journal.pone.0207120)
Supplement: S1 Table — (DOCX) [file pone.0207120.s001.docx]

**S1 Table. Summary of Patients From RCTs Included in Virtual Lab 2.0, by Pregabalin Dose (Table 1 With Supplemental Notes).**

|  | **5/6 Weeks Studies*** | | **12/13 Weeks Studies**** | | **Total RCT Patients** | |
| --- | --- | --- | --- | --- | --- | --- |
| **Pregabalin dose** | ***n*** | **% of total** | ***n*** | **% of total** | ***n*** | **% of total** |
| Flexible dose^a^ | 0 | 0.0 | 83 | 6.3 | 83 | 6.3 |
| Flexible adjusted dose^b^ | 0 | 0.0 | 193 | 14.6 | 193 | 14.6 |
| 75 mg/day | 59 | 4.5 | 0 | 0.0 | 59 | 4.5 |
| 150 mg/day | 69 | 5.2 | 74 | 5.6 | 143 | 10.8 |
| 300 mg/day | 124 | 9.4 | 297 | 22.5 | 421 | 31.9 |
| 600 mg/day | 129 | 9.8 | 292 | 22.1 | 421 | 31.9 |
| **Total** | **381** | **28.9** | **939** | **71.1** | **1320** | **100.0** |

*n*, number of patients; RCT, randomized controlled trial.

^a^ Patients with 1–4 weeks escalation phase and 8–11 weeks maintenance (Protocol 1008-155).

^b^ Patients with 6 weeks escalation phase and 6 weeks maintenance (Protocol A0081030).

*** Study 1008-014 [16] conducted Mar 1998–Mar 1999 in US/Canada, DB-RCT with 1 week baseline, 2 weeks dose escalation, 4 weeks maintenance with placebo, pregabalin 150 and 600 mg/day doses; Study 1008-029 [15] conducted Apr 1998–Jun 1999 in US, DB-RCT with 1 week baseline, 1 week dose escalation, 4 weeks maintenance with placebo, pregabalin 75, 300, and 600 mg/day doses; Study 1008-131 [17] conducted Dec 1999–May 2000 in US, DB-RCT with 1 week baseline, 6 weeks maintenance with placebo, and pregabalin 300 mg/day dose.

** Study 1008-149 [18] conducted Nov 2000–May 2002 in Australia/Europe/South Africa, DB-RCT with 1 week baseline, 1 week dose escalation, 11 weeks maintenance with placebo, pregabalin 150, 300, and 600 mg/day doses; Study 1008-155 (A0081049) [14] conducted Jul 2001–Dec 2002 in Europe, DB-RCT with 1 week baseline, 1–4 weeks dose escalation, 8–11 weeks maintenance with placebo, pregabalin 150–600 mg/day flexible dosing, and pregabalin 600 mg/day doses; Study A0081030 [20] conducted Jan 2005–Apr 2006 in Asia/Latin America/Middle East, DB-RCT with 1 week baseline, 6 weeks dose escalation, 6 weeks maintenance, 1 week withdrawal with placebo, pregabalin 150–600 mg/day flexible dosing; A0081060 [19] conducted Sep 2004–Oct 2005 in US, DB-RCT with 1 week baseline, 1 week dose escalation, 12 weeks maintenance with placebo, pregabalin 600 mg/day dose; A0081071 (data on file) conducted May 2005–May 2007 in US, DB-RCT with 1–2 weeks baseline, 1 week dose escalation, 12 weeks maintenance, 1 week withdrawal with placebo, pregabalin 300 and 600 mg/day doses; A0081163 [21] conducted Oct 2007–Mar 2009 in Japan, DB-RCT with 1 week baseline, 1 week dose escalation, 12 weeks maintenance, 1 week withdrawal with placebo, pregabalin 300 and 600 mg/day doses.
