# Supplementary material for: Using time series analysis approaches for improved prediction of pain outcomes in subgroups of patients with painful diabetic peripheral neuropathy
Source: PLoS One. 2018 Dec 6;13(12):e0207120. doi: 10.1371/journal.pone.0207120 (PMC6283469; doi:10.1371/journal.pone.0207120)
Supplement: S2 Table — (DOCX) [file pone.0207120.s002.docx]

**S2 Table: Comparison of LASSO, Adaptive LASSO, and Elastic Net Regressions**

|  |  | **CLUSTER** | | | | | |
| --- | --- | --- | --- | --- | --- | --- | --- |
|  |  | **1** | **2** | **3** | **4** | **5** | **6** |
| **Adj R-sq** | **LASSO** | 0.9799 | 0.9797 | 0.9842 | 0.9838 | 0.9856 | 0.9832 |
|  | **adaptive LASSO** | 0.9802 | 0.98370 | 0.98420 | 0.98390 | 0.98520 | 0.98410 |
|  | **elastic net** | 0.97970 | 0.98370 | 0.98420 | 0.98380 | 0.98560 | 0.98320 |
| **RMSE** | **LASSO** | 0.40219 | 0.40420 | 0.42600 | 0.39856 | 0.38564 | 0.39166 |
|  | **adaptive LASSO** | 0.39871 | 0.46031 | 0.42617 | 0.39751 | 0.39179 | 0.38978 |
|  | **elastic net** | 0.40420 | 0.46054 | 0.42600 | 0.39856 | 0.38564 | 0.39124 |
| **Degree of freedom (# of variables)** | **LASSO** | 22 | 24 | 24 | 30 | 27 | 23 |
|  | **adaptive LASSO** | 15 | 18 | 20 | 26 | 9 | 33 |
|  | **elastic net** | 24 | 25 | 24 | 30 | 27 | 27 |
