# Supplementary material for: Using time series analysis approaches for improved prediction of pain outcomes in subgroups of patients with painful diabetic peripheral neuropathy
Source: PLoS One. 2018 Dec 6;13(12):e0207120. doi: 10.1371/journal.pone.0207120 (PMC6283469; doi:10.1371/journal.pone.0207120)
Supplement: S3 Table — (DOCX) [file pone.0207120.s003.docx]

**S3 Table. CEM Results.**

| **Cluster** | **Patients by Cluster, *n*** | | | **Global Imbalance** | | **Reduction in Global Imbalance**  **After CEM (%)** |
| --- | --- | --- | --- | --- | --- | --- |
|  | **OS Alone** | | **OS + RCT in Matched Dataset**  **After CEM, *n*** |  |  |  |
|  | **Before CEM** | **After CEM** |  | **Before CEM** | **After CEM** |  |
| 1 | 579 | 431 | 542 | 0.66 | 0.32 | 51.5 |
| 2 | 283 | 189 | 487 | 0.69 | 0.34 | 50.7 |
| 3 | 626 | 437 | 473 | 0.58 | 0.31 | 46.6 |
| 4 | 473 | 266 | 419 | 0.77 | 0.36 | 53.2 |
| 5 | 195 | 127 | 470 | 0.76 | 0.33 | 56.7 |
| 6 | 486 | 316 | 452 | 0.64 | 0.32 | 50.0 |
| **Total** | **2642** | **1766** | **2843** | 0.68 (mean) | 0.33 (mean) | 51.5 (mean) |

CEM, coarsened exact matching; OS, observational study; RCT, randomized controlled trial.
