# Supplementary material for: Using time series analysis approaches for improved prediction of pain outcomes in subgroups of patients with painful diabetic peripheral neuropathy
Source: PLoS One. 2018 Dec 6;13(12):e0207120. doi: 10.1371/journal.pone.0207120 (PMC6283469; doi:10.1371/journal.pone.0207120)
Supplement: S4 Table — (DOCX) [file pone.0207120.s004.docx]

**S4 Table. Regression Model Input Variables and Resulting Regression Coefficients by Cluster for the Calibration Dataset Before Regularization.**

| **Model Input Variables** | **Final Output Regression Coefficients, by Cluster^a^** | | | | | |
| --- | --- | --- | --- | --- | --- | --- |
|  | **1** | **2** | **3** | **4** | **5** | **6** |
| y-intercepts for regression models, not variables | 0.0183 | 0.0148 | 1.0988 | 0.0429 | 0.0867 | 0.2490 |
| Age cohort (**x9**) | - | - | - | - | -0.0184 | - |
| Insulin use (**x10**) | - | - | -0.5298 | - | - | - |
| Pain score (t-1)^b^ (**x1**) | 0.7317 | 0.8269 | 0.7928 | 0.8082 | 0.7895 | 0.7689 |
| Pain score (t-2)^c^ (**x2**) | 0.0371 | 0.0014 | 0.0233 | 0.0403 | -0.0416 | 0.0410 |
| Pain score (t-3)^d^ (**x3**) | - | - | - | -0.0772 | - | - |
| PRSI score (t)^e^ (**x4)** | 0.5898 | 0.4522 | 0.5535 | 0.5211 | 0.6238 | 0.6181 |
| PRSI score (t-1)^b^ (**x5**) | -0.4080 | -0.3380 | -0.4104 | -0.3946 | -0.4396 | -0.4985 |
| PRSI score (t-2)^c^ (**x6**) | - | - | - | 0.0312 | - | - |
| Dose (t-1)^b^ (**x7**) | -0.0007 | -0.0001 | -0.0004 | -0.0002 | -0.0002 | -0.0005 |
| Dose (t-2)^c^ (**x8**) | 0.0008 | 0.0003 | 0.0006 | 0.0004 | 0.0002 | 0.0005 |
| General feeling: full of energy (t-1)^b^ (**x11**) | - | - | - | -0.0964 | -0.0381 | - |
| General feeling: full of energy (t-2)^c^ (**x12**) | - | - | - | 0.1128 | 0.0809 | - |
| General feeling: sad and discouraged (t-1)^b^ (**x13**) | -0.0468 | -0.0615 | -0.0934 | - | - | -0.0833 |
| General feeling: sad and discouraged (t-2)^c^ (**x14**) | 0.0498 | 0.0495 | 0.0819 | - | - | 0.0605 |
| Model performance measures applied | Performance, by cluster | | | | | |
|  | 1 | 2 | 3 | 4 | 5 | 6 |
| Likelihood ratio *P*-value | < 0.0001 | < 0.0001 | < 0.0001 | < 0.0001 | < 0.0001 | < 0.0001 |
| *Adjusted R^2^* | 0.90 | 0.91 | 0.93 | 0.91 | 0.93 | 0.91 |
| Root mean square error | 0.44 | 0.48 | 0.45 | 0.41 | 0.42 | 0.42 |
| Observed vs. estimated responders (Student’s *t* test *P-*value)^f^ | 1.00 | 1.00 | 1.00 | 1.00 | 1.00 | 0.99 |

PRSI, pain-related sleep interference.

^a^ The first number in each column is the regression intercept value. Blank spaces in columns indicate that the associated row variable was not a predictor in the final model for that cluster.

^b^ (t-1) indicates 1 week before prediction.

^c^ (t-2) indicates 2 weeks before prediction.

^d^ (t-3) indicates 3 weeks before prediction.

^e^ (t) indicates the same week of the prediction.

Given the time series of pain scores, a linear regression model with lagged variables as inputs provides a simple pathway for incorporating time series data. The regression model inputs were assigned unique variable names, x1-x14, and are represented in the cluster-specific regressions below:

**Equations for the regressions** (where ‘y’ is the fitted pain score) both for H.1

**CLUSTER 1:** y = 0.0183 + 0.7317**x1** + 0.0371**x2** + 0.5898**x4** - 0.4080**x5** - 0.0007**x7** + 0.0008**x8 -** 0.0468**x13 +** 0.0498**x14**

**CLUSTER 2:** y = 0.0148 + 0.8269**x1** + 0.0014**x2** + 0.4522**x4** - 0.3380**x5** - 0.0001**x7** + 0.0003**x8** - 0.0615**x13** + 0.0495**x14**

**CLUSTER 3:** y = 1.0988 + 0.7928**x1** + 0.0233**x2** + 0.5535**x4** - 0.4104 **x5** - 0.0004 **x7** + 0.0006 **x8** - 0.5298**x10** - 0.0934**x13** + 0.0819 **x14**

**CLUSTER 4:** y = 0.0429 + 0.8082**x1** + 0.0403**x2** - 0.0772**x3** + 0.5211**x4** - 0.3946**x5** + 0.0312**x6**- 0.0002**x7** + 0.0004**x8** - 0.0964**x11 +** 0.1128**x12**

**CLUSTER 5:** y = 0.0867 + 0.7895**x1** - 0.0416**x2** + 0.6238**x4** - 0.4396**x5** - 0.0002**x7** + 0.0002**x8** - 0.0184**x9** - 0.0381**x11** + 0.0809**x12**

**CLUSTER 6:** y = 0.2490 + 0.7689**x1** + 0.0410**x2** + 0.6181**x4** - 0.4985**x5** - 0.0005**x7** + 0.0005**x8** - 0.0833**x13** + 0.0605**x14**

^f^ The regressions estimate pain score, but we also want to be able to identify whether that patient is a responder at different thresholds (eg, 50% or 30% reduction in pain score). Hence, we wanted to confirm estimation of responder level based on the regression for pain score.
