# Supplementary material for: Using time series analysis approaches for improved prediction of pain outcomes in subgroups of patients with painful diabetic peripheral neuropathy
Source: PLoS One. 2018 Dec 6;13(12):e0207120. doi: 10.1371/journal.pone.0207120 (PMC6283469; doi:10.1371/journal.pone.0207120)
Supplement: S5 Table — (DOCX) [file pone.0207120.s005.docx]

**S5 Table. Change in Responder Status at 6 Weeks Compared With the End of the Study.**

| **Study** | **Non-responder at Week 6 but Became a Responder at Week 12 or 13**  **n (%)** | **Responder at Week 6 but Became a Non-responder at Week 12 or 13**  **n (%)** | **Did Not Change Responder Status Between Week 6 and Week 12 or 13**  **n (%)** | **5- or 6-Week Studies** | **Total** |
| --- | --- | --- | --- | --- | --- |
| 1008-014 |  |  |  | 135 |  |
| 1008-029 |  |  |  | 188 |  |
| 1008-131 |  |  |  | 58 |  |
| 1008-149 | 26 (12.2%) | 17 (7.9%) | 171 (79.9%) |  | 214 |
| 1008-155 | 19 (12.4%) | 7 (4.6%) | 127 (83.0%) |  | 153 |
| A0081030 | 17 (8.8%) | 13 (6.7%) | 163 (84.5%) |  | 193 |
| A0081060 | 2 (4.7%) | 4 (9.3%) | 37 (86.0%) |  | 43 |
| A0081071 | 28 (14.7%) | 20 (10.5%) | 143 (74.9%) |  | 191 |
| A0081163 | 14 (9.7%) | 10 (6.9%) | 121 (83.4%) |  | 145 |
| A0081061 |  |  |  | 2642 |  |
| Total | **106** (11.3% of 12- or 13-week studies) | **71**  (7.6% of 12- or 13-week studies) | **762**  (81.2% of 12- or 13-week studies) | 3023 | **939** |
