# Supplementary material for: Using time series analysis approaches for improved prediction of pain outcomes in subgroups of patients with painful diabetic peripheral neuropathy
Source: PLoS One. 2018 Dec 6;13(12):e0207120. doi: 10.1371/journal.pone.0207120 (PMC6283469; doi:10.1371/journal.pone.0207120)
Supplement: S6 Table — (DOCX) [file pone.0207120.s006.docx]

**S6 Table. Regression Model Performance by Cluster: Two-Sample *t*-Test^a^.**

|  | **Cluster 1** | **Cluster 2** | **Cluster 3** | **Cluster 4** | **Cluster 5** | **Cluster 6** |
| --- | --- | --- | --- | --- | --- | --- |
| Calibration (1766 patients) | 1.00 | 1.00 | 1.00 | 1.00 | 1.00 | 0.99 |
| Validation  (876 patients) | 0.77 | 0.64 | 1.00 | 0.71 | 0.77 | 0.49 |

^a^ As the goal is statistical similarity, *P*-values closer to 1 are desirable.
