# Supplementary figures and images for: Using time series analysis approaches for improved prediction of pain outcomes in subgroups of patients with painful diabetic peripheral neuropathy
Source: PLoS One. 2018 Dec 6;13(12):e0207120. doi: 10.1371/journal.pone.0207120 (PMC6283469; doi:10.1371/journal.pone.0207120)

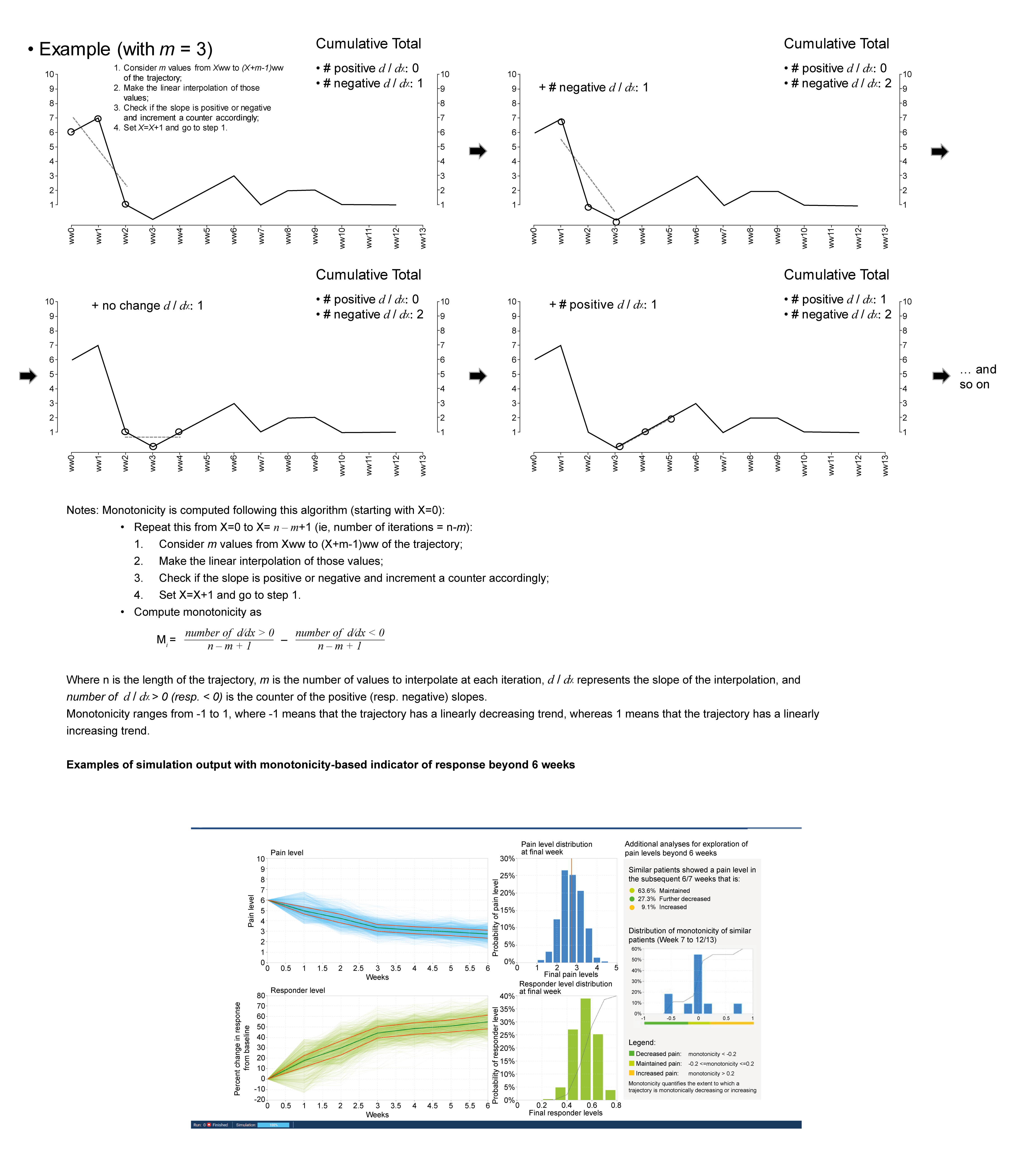

Supplement: S1 Fig — (TIF) [file pone.0207120.s007.tif]

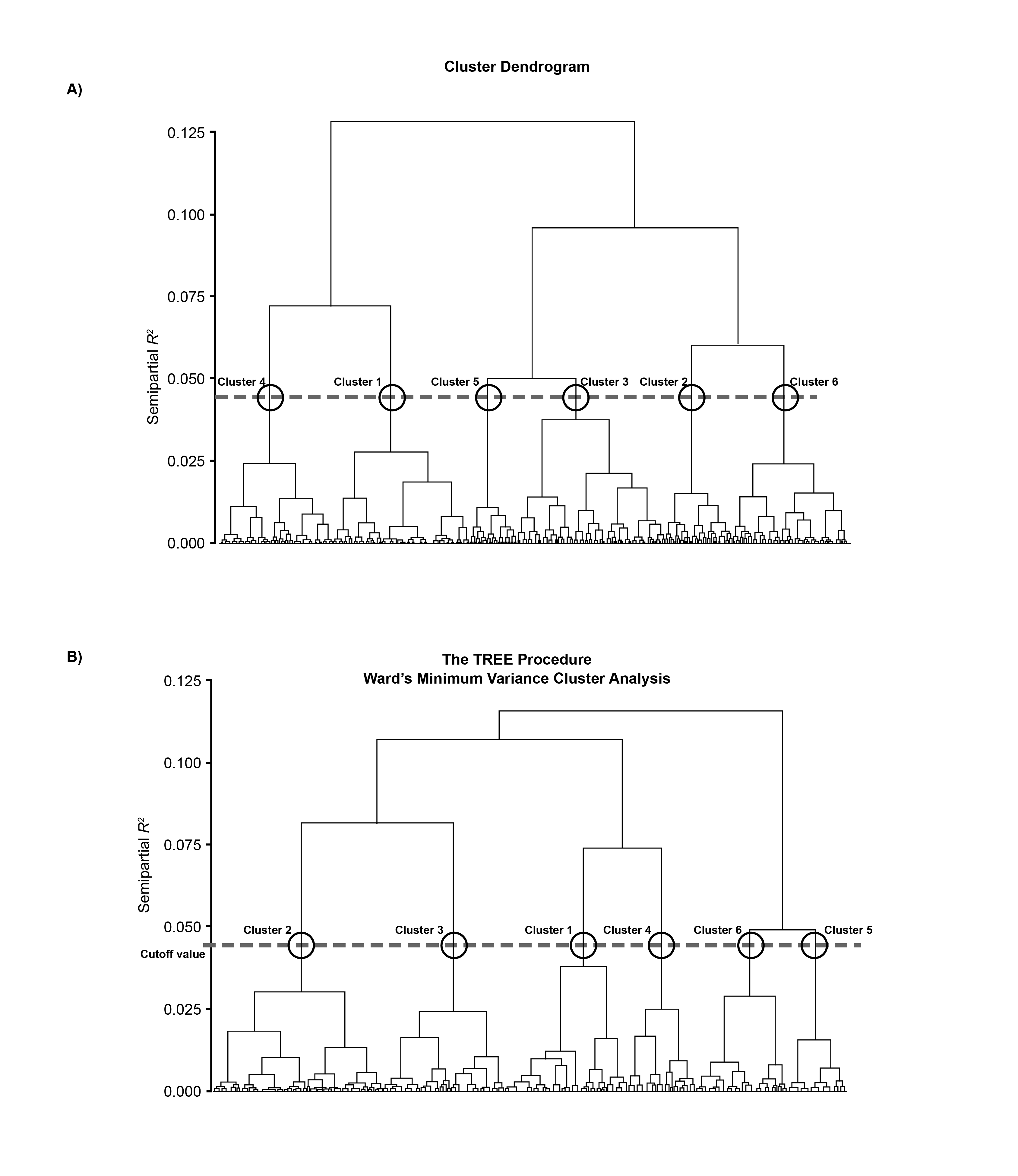

Supplement: S2 Fig — (A) Virtual Lab 2.0. (B) Virtual Lab 1.0. (TIF) [file pone.0207120.s008.tif]
